# Validation and Comparison of Aneurysmal Subarachnoid Hemorrhage Grading Scales in Angiogram-Negative Subarachnoid Hemorrhage Patients

**DOI:** 10.1155/2020/9707238

**Published:** 2020-02-28

**Authors:** Yuanjian Fang, Shenbin Xu, Jianan Lu, Haijian Wu, Jingwei Zheng, Cameron Lenahan, Yang Cao, Sheng Chen, Zefeng Wang, Jianmin Zhang

**Affiliations:** ^1^Department of Neurosurgery, The Second Affiliated Hospital, School of Medicine, Zhejiang University, Hangzhou, Zhejiang, China; ^2^Center for Neuroscience Research, Loma Linda University School of Medicine, Loma Linda, CA, USA; ^3^Burrell College of Osteopathic Medicine, Las Cruces, NM, USA; ^4^Brain Research Institute, Zhejiang University, Hangzhou, Zhejiang, China; ^5^Collaborative Innovation Center for Brain Science, Zhejiang University, Hangzhou, Zhejiang, China

## Abstract

Numerous grading scales have been proposed to predict the outcome of aneurysmal subarachnoid hemorrhage (SAH); however, these have not been validated in angiogram-negative SAH patients. In this study, we aim to validate and compare the aneurysmal SAH grading scales in angiogram-negative SAH patients. There were 190 angiogram-negative SAH patients analyzed from January 2014 to December 2015. The outcomes were measured by delayed cerebral ischemia (DCI) and poor outcome (defined as modified Rankin Scale (mRS) 3-6 or 4-6). The predictive performance of the grading scales was assessed via evaluation of distribution, trend, association, and discrimination. In regard to the distribution, none of the patients were categorized as HAIR 8 and SAH score 8. Both grading scales indicated a significant trend between scores and outcome (*P* < 0.05), and association with the outcome (OR > 1). The modified Fisher Scale (mFS), World Federation of Neurosurgical Societies scale (WFNS), and combined scores VASOGRADE and HAIR showed good predictive accuracy (area under the curve (AUC) > 0.750) for DCI. The predictive accuracy in each scale performed well in predicting poor outcome, with the exception of mFS and the Subarachnoid hemorrhage Early Brain Edema Score (SEBES). However, the mFS performed with increased accuracy when predicting mRS 4-6. The VASOGRADE, HAIR, and WFNS may be valuable prognostic tools for predicting both DCI and poor outcome. The mFS can be applicable for predicting DCI and mRS 4-6. The SAH score and the Hunt-Hess were also optimal for predicting poor outcome. The predictive performance of SEBES was relatively poor compared to the other scales.

## 1. Introduction

Angiogram-negative subarachnoid hemorrhage (SAH), accounting for 15-20% of SAH, was considered a special type of spontaneous SAH with a benign progression and better outcome compared to aneurysmal SAH [1, 2]. However, angiogram-negative SAH still has a definite incidence of delayed cerebral ischemia (DCI) and poor outcome, particularly in patients with a nonperimesencephalic bleeding pattern [1]. More than 40 prognostic grading scores [3], including the Hunt-Hess (HH) [4], the World Federation of Neurosurgical Societies scale (WFNS) [5], the modified Fisher Scale (mFS) [6], the Subarachnoid hemorrhage Early Brain Edema Score (SEBES) [7], the VASOGRADE [8], the HAIR [9], and the SAH score [10], were proposed to guide the clinical treatment after SAH. However, none of these were initially designed for use in angiogram-negative SAH patients. These grading scales were derived and validated in the aneurysmal SAH patients but were also commonly used in the clinical assessment and prognosis of angiogram-negative SAH patients. The effectiveness of clinical, radiological, or combined scores has rarely been validated in patients with angiogram-negative SAH. Herein, we designed this study to validate and compare the predictive performance of aneurysmal SAH grading scales in patients with negative angiograms. An optimal selection of grading scales may assist neurologists in making informed decisions, allowing for improved care and treatment in angiogram-negative patients.

## 2. Methods

### 2.1. Study Population

Our study retrospectively reviewed past clinical records and imaging data of 1,119 spontaneous SAH patients in The Second Affiliated Hospital of Zhejiang University School of Medicine between January 2014 and December 2015 with approval from the local Institutional Review Board. No patient consent was required in our study, as our Institutional Review Board had approved full waiver of consent. All SAH patients were screened by computed tomography angiography (CTA) at admission, followed by emergent digital subtraction angiography (DSA) examination within 72 hours. The inclusion criteria were derived from the definition of angiogram-negative SAH, as previously described in a study with patients lacking a definitive causative lesion on CT angiography and DSA [1]. Patients were excluded if they sought care more than 3 days after the onset of SAH, if they had a history of trauma or previous brain injury (chronic changes depicted on CT imaging), if the patient had serious comorbidities prior to SAH onset (i.e., severe coagulation disorders and uncontrolled arrhythmia), and lastly, if patients' radiological data were unavailable. Furthermore, we reviewed the baseline characteristics, such as age, sex, history of smoking and drinking, hypertension, hyperlipidemia, diabetes, and heart disease. Prognostic grading scales were divided into clinical, radiological, and combined scores. Clinical scores included the WFNS and the HH, and these were obtained by reviewing the medical records at admission. Radiological scores included the mFS and the SEBES, and these were scored by two double-blinded neurologists based on the CT image at admission. An independent neurologist was applied if there was a lack of consensus among the former two neurologists. The combined scores, including the VASOGRADE, HAIR, and the SAH score, were scored according to clinical and radiological data. The length of hospital stay (LOS) and SAH-related complications were also recorded in the baseline characteristics.

### 2.2. Patient Management

All patients were treated according to the Neurocritical Care Society and American Heart Association SAH guidelines [11, 12]. Nimodipine was administered to prevent cerebral vasospasm, and euvolemia was maintained in all patients via intravenous hydration. Hemodynamic values were monitored via electrocardiogram while the patients were admitted.

### 2.3. Outcome Measures

Two outcome measures were used, including DCI and poor outcome. DCI was defined as symptomatic vasospasm or emergence of a new infarction on CT or magnetic resonance imaging (MRI). Symptomatic vasospasm was diagnosed as the development of new focal neurological signs or deterioration of consciousness, which excluded other definite causes [13]. Each patient underwent routine CT examination prior to being discharged.

Poor outcome was measured by the modified Rankin Scale (mRS) at three months after discharge [14] and recorded by outpatient records or telephone interview. The mRS was divided into two binary outcome categories: (1) poor outcome—mRS 3-6, favorable outcome—mRS 0-2; (2) poor outcome—mRS 4-6, favorable outcome—mRS 0-3.

### 2.4. Statistical Analysis

SPSS 22.0 (SPSS Institute, Chicago, IL, USA) and MedCalc Statistical Software version 18.2.1 (MedCalc Software bvba, Ostend, Belgium; http://www.medcalc.org; 2018) were used for statistical analysis. *P* < 0.05 was considered statistically significant. Continuous variables were expressed as means ± standard deviations (SD). Categorical variables were expressed as frequencies with percentages. Predictive performance of grading scales was compared by distribution, trend, odds ratio (OR), and discrimination [15]. The trends between scores in grading scale and prognostic indicators, as well as OR values, were analyzed by binary logistic regression analysis. Discriminations were presented as receiver operating characteristic (ROC) curves, and were compared with the area under the ROC curve (AUC) by using the Delong test. AUC > 0.750 was considered favorable predictive accuracy [16].

## 3. Results

### 3.1. Patient Characteristic

**Figure 1:**
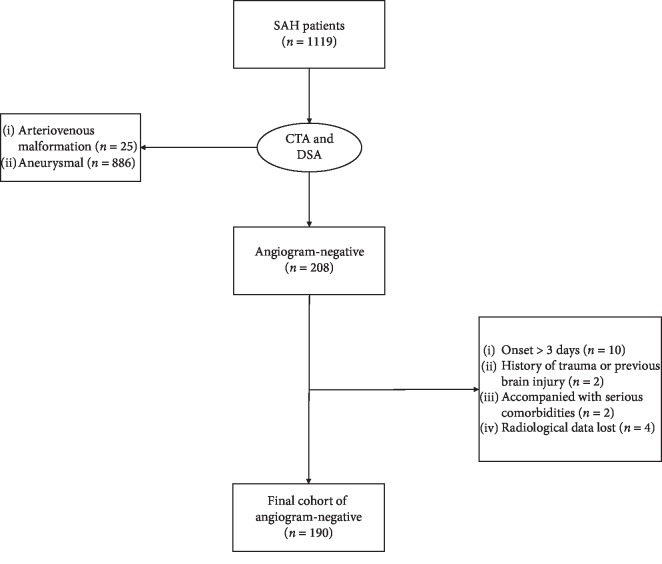
Flow chart of angiogram-negative SAH patients. Abbreviations: CTA—computed tomography angiography; DSA—digital subtraction angiography; SAH—subarachnoid hemorrhage.

A total of 208 SAH patients had negative angiogram at admission, with 190 patients included in the final cohort for analysis. The remaining 18 patients were excluded as follows: 10 patients who presented to the hospital more than 3 days after SAH onset, 2 patients with a history of trauma, 2 patients with serious arrhythmia prior to SAH onset, and 4 patients without CT image at admission (Figure 1).

Among 190 angiogram-negative patients, the ages ranged from 24 to 85 years (mean 57.5 ± 11.8 years) and 96 (50.5%) were male. Patients' characteristics are presented in Table 1. A total of 11 (5.8%) patients suffered from DCI, 9 suffered from both symptomatic vasospasm and delayed cerebral infarction, and 2 suffered from only delayed cerebral infraction. There were 3 new infarctions found by both MRI and CT, but those found in the remaining 8 patients were found by CT. Lastly, 20 (10.5%) patients had an unfavorable outcome with mRS 3 to 6, and 13 (6.8%) had an unfavorable outcome with mRS 4 to 6.

### 3.2. Distribution of Grading Scales

**Figure 2:**
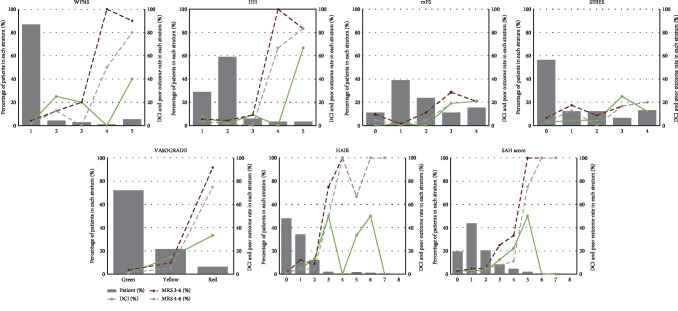
Distribution and outcome rate of grading scales. Abbreviations: DCI—delayed cerebral ischemia; HH—Hunt-Hess; mFS—modified Fisher Scale; mRS—modified Rankin Scale; SAH—subarachnoid hemorrhage; SEBES—Subarachnoid hemorrhage Early Brain Edema Score; WFNS—World Federation of Neurosurgical Societies.

In clinical scales, most patients had a WFNS grade of 1 (86.8%) and an HH grade of 2 (58.9%). In radiological scales, 56.3% of patients had a SEBES grade of 0, while mFS had a relatively equal patient distribution in each grade. As for the combined scales, VASOGRADE green (72.1%) had the highest percentage of patients. More than 80% of patients had HAIR grades of 0 (47.9%) and 1 (34.2%), but a reduced number of patients had a HAIR grade of 8. Similarly, most patients had a SAH score grade of 1 (43.7%) but there was a reduced number of patients in grade 8 (Figure 2).

### 3.3. Predictive Performance for DCI

**Figure 3:**
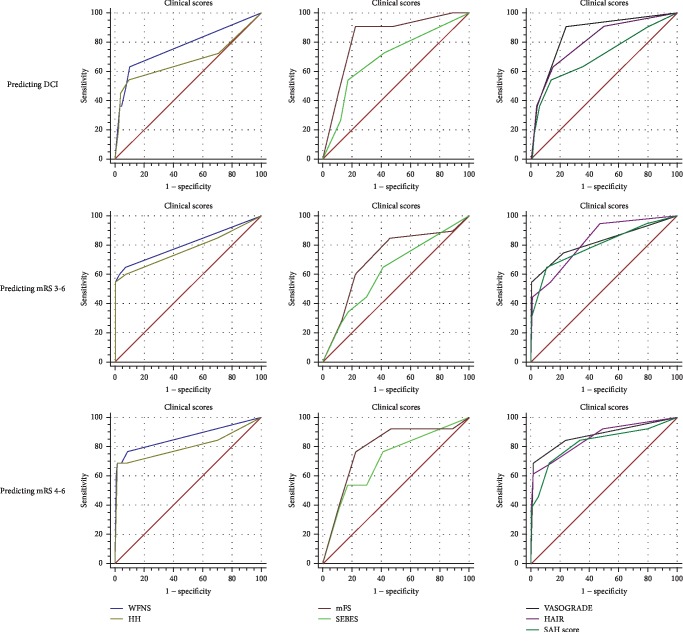
ROCs of scales. Abbreviations: DCI—delayed cerebral ischemia; HH—Hunt-Hess; mFS—modified Fisher Scale; mRS—modified Rankin scale; SAH—subarachnoid hemorrhage; SEBES—Subarachnoid hemorrhage Early Brain Edema Score; WFNS—World Federation of Neurosurgical Societies.

The VASOGRADE had the highest OR values (OR = 6.916, 95%CI = 2.840‐16.844), with the mFS (OR = 3.193, 95%CI = 1.684‐6.053), HH (OR = 2.431, 95%CI = 1.461‐4.044), WFNS (OR = 2.170, 95%CI = 1.493‐3.154), HAIR (OR = 1.849, 95%CI = 1.315‐2.601), SAH score (OR = 1.735, 95%CI = 1.192‐2.525), and the SEBES (OR = 1.587, 95%CI = 1.092‐2.307) following in succession (Table 2). All scales, including the WFNS (*P* for trend < 0.001), HH (*P* for trend < 0.001), mFS (*P* for trend < 0.001), SEBES (*P* for trend < 0.015), VASOGRADE (*P* for trend < 0.001), the HAIR (*P* for trend < 0.001), and the SAH score (*P* = 0.004) showed a strong trend between increasing scores and DCI rate (Table 2 and Figure 2). The VASOGRADE (AUC = 0.858, 95%CI = 0.800‐0.904), mFS (AUC = 0.839, 95%CI = 0.779‐0.888), HAIR (AUC = 0.809, 95%CI = 0.746‐0.863), and WFNS (AUC = 0.773, 95%CI = 0.707‐0.831) showed favorable predictive accuracy (Table 2 and Figure 3), despite a lack of statistical significance being found in the statistical comparison of AUC values (Table 3).

### 3.4. Predictive Performance for Poor Outcome

For predicting mRS 3-6, the OR values of VASOGRADE (OR = 11.542, 95%CI = 7.071‐26.940) were double those of the other scales. The following scales are listed in descending order, according to their respective OR values: HH (OR = 4.632, 95%CI = 2.595‐8.302), WFNS (OR = 3.810, 95%CI = 2.375‐6.113), HAIR (OR = 3.081, 95%CI = 1.906‐4.981), SAH score (OR = 2.846, 95%CI = 1.901‐4.262), mFS (OR = 1.813, 95%CI = 1.242‐2.645), and SEBES (OR = 1.336, 95%CI = 1.002‐1.781) (Table 2). All scales, including the WFNS (*P* for trend < 0.001), HH (*P* for trend < 0.001), mFS (*P* for trend < 0.002), SEBES (*P* for trend < 0.049), the VASOGRADE (*P* for trend < 0.001), HAIR (*P* for trend < 0.001), and the SAH score (*P* < 0.001) showed a strong trend between increasing scores and poor outcome rate (Table 2 and Figure 2). Meanwhile, the clinical scales and combined scales showed a good predictive accuracy. The order of AUC values are as follows: HAIR (AUC = 0.833, 95%CI = 0.772‐0.883), VASOGRADE (AUC = 0.823, 95%CI = 0.761‐0.874), WFNS (AUC = 0.807, 95%CI = 0.744‐0.861), SAH score (AUC = 0.806, 95%CI = 0.742‐0.860), HH (AUC = 0.774, 95%CI = 0.708‐0.831), mFS (AUC = 0.712, 95%CI = 0.643‐0.776), and the SEBES (AUC = 0.629, 95%CI = 0.556‐0.697) (Table 2). The AUC of the SEBES was significantly lower than the clinical and radiological scales (*P* = 0.022 vs. WFNS; *P* = 0.013 vs. VASOGRADE; *P* = 0.009 vs. HAIR; *P* = 0.048 vs. SAH score) (Table 3).

When predicting mRS 4-6, the VASOGRADE (OR = 17.015, 95%CI = 5.651‐51.234) still maintained the highest OR value. The order of OR values remained the same in regard to predicting mRS 3-6: HH (OR = 4.963, 95%CI = 2.681‐9.186), WFNS (OR = 3.686, 95%CI = 2.346‐5.792), HAIR (OR = 3.385, 95%CI = 2.000‐5.742), SAH score (OR = 2.806, 95%CI = 1.799‐4.374), mFS (OR = 2.439, 95%CI = 1.242‐2.645), and the SEBES (OR = 1.631, 95%CI = 1.151‐2.313). All scales displayed a trend between increasing scores and poor outcome rates: the WFNS (*P* for trend < 0.001), HH (*P* for trend < 0.001), mFS (*P* for trend < 0.001), SEBES (*P* for trend < 0.006), VASOGRADE (*P* for trend < 0.001), HAIR (*P* for trend < 0.001), and the SAH score (*P* < 0.001) (Table 2 and Figure 2). All scales but the SEBES (AUC = 0.711, 95%CI = 0.641‐0.775) showed favorable predictive accuracy. The order of AUC values were as follows: the VASOGRADE (AUC = 0.879, 95%CI = 0.824‐0.922), WFNS (AUC = 0.865, 95%CI = 0.808‐0.910), HAIR (AUC = 0.861, 95%CI = 0.803‐0.907), SAH score (AUC = 0.829, 95%CI = 0.768‐0.880), and the mFS (AUC = 0.789, 95%CI = 0.724‐0.845) (Table 2). No statistical difference was found between each grading scale, though VASOGRADE was more statistically accurate than SEBES (*P* = 0.034) and HH (*P* = 0.029) (Table 3).

## 4. Discussion

This study validated and compared the predictive performance of aneurysmal SAH prognostic grading scales in negative-angiogram SAH patients. The effectiveness of each scale varied in predicting different prognostic indicators. Each scale was significantly associated with DCI and presented a trend among rising scores and DCI rates. However, only the VASOGRADE, HAIR, mFS, and WFNS showed the good predictive accuracy (AUC > 0.750) of DCI (Table 2). In addition, each scale showed an association and good trend with poor outcome as measured by mRS. The predictive accuracy excelled in all scales, with the exception of the radiological scales (mFS and SEBES) for predicting mRS 3-6. However, the mFS had increased accuracy when predicting mRS 4-6 (Table 2).

The VASOGRADE maintained a favorable and leading predictive performance in each outcome category of our study. The VASOGRADE simply combined the WFNS and the mFS to stratify the DCI risk into green, yellow, and red. Furthermore, it was validated by a cohort of 746 aneurysmal SAH patients, which presented an acceptable discrimination (AUC = 0.630) and calibration for the prediction of DCI [8]. However, the discrimination of VASOGRADE for predicting DCI and poor outcome in angiogram-negative SAH is quite higher than the aneurysmal SAH in our study, showing an eligible predictive performance for angiogram-negative SAH patients. In contrast, HAIR seems more complicated than VASOGRADE, which was a typical risk stratification scale derived from multivariate logistic regression analysis ranging from 0 to 8. It combined variables, such as HH, age, and intraventricular hemorrhage and rebleed. It then assigns a corresponding score to predict the in-hospital mortality of SAH patients. HAIR did not distinguish between aneurysmal and nonaneurysmal SAH in the derivation and validation cohorts. They claimed that HAIR can also be utilized in the angiogram-negative SAH patients, and the AUC value in their study is quite high (AUC = 0.910) [9]. However, as we measured HAIR, we found that it does have favorable predictivity compared to the other scales, but it was not better than its original AUC value. Similar to HAIR, the SAH score used multivariate logistic regression analysis in aneurysmal SAH patients' risk factors to build a risk score that combined the Glasgow Coma Scale, age, and comorbidities for prediction of mortality [10]. In this study, we found that the SAH score also can be a viable predictive scale for predicting poor outcomes (AUC = 0.806 for predicting mRS 3-6 and AUC = 0.829 for predicting mRS 4-6), but not DCI (AUC = 0.710) of angiogram-negative patients.

As a combined scale, the VASOGRADE, HAIR, and SAH scores were originally designed to avoid the limitations of a single score [8, 9]. Those single scales mainly focused on the clinical symptoms or radiological images, which may potentially misdiagnose patients presenting with serious radiological imaging and mild clinical symptoms. However, it was found that the combined grading systems showed no superiority compared with the clinical score in predicting DCI and unfavorable patient outcome in aneurysmal SAH patients [15]. Interestingly, the dominance of VASOGRADE in predicting DCI and poor outcome was shown in the angiogram-negative SAH patients of our study. Additionally, there was statistical significance when compared with SEBES for predicting poor outcome. We speculate that the treatment of aneurysmal SAH, such as clipping or coiling, and the existence of differences among the analysis cohorts, particularly with respect to the varying ratios of the percentage of clipping or coiling treatments used with each respective study, may interfere with the predictability of scales.

SEBES is a recently proposed radiological scale that emphasizes early brain changes that emerge on CT imaging. It assesses early brain edema and assigns points ranging from 0 to 4, according to the visible sulci and disruption of the gray-white matter junction at two predetermined levels in each hemisphere to predict the DCI and poor outcome of aneurysmal patients. After validation in 164 aneurysmal SAH patients, SEBES showed a good AUC value (0.790 for predicting DCI and 0.780 for predicting poor outcome) [7], but the predictive accuracy was relatively unremarkable in the angiogram-negative in our study, especially when compared to the combined scales of VASOGRADE. As a newly proposed radiological scale, we suggest further studies with larger cohorts to validate the effectiveness of SEBES in aneurysmal and nonaneurysmal patients.

A recent study compared grading scales in 423 aneurysmal SAH patients and found that the mFS was inferior to the clinical scales for predicting unfavorable patient outcomes due to cerebral infarction (*P* < 0.05 for AUC comparison) [15]. However, while predicting poor outcomes in angiogram patients, the mFS performed more poorly than the combined and clinical scales but did not reach the statistical baseline in our study. Conversely, it should be mentioned that the predictive performance (second OR and AUC value) of mFS for DCI is quite different compared to the poor outcome in our patient cohort. Lastly, we speculate that the mFS may still be a viable option for predicting DCI in angiogram-negative patients, as it was originally designed in aneurysmal patients [6].

### 4.1. Limitations

There are some limitations present within our study. First, our study is a retrospective and single-center study, which may introduce potential bias in patient characteristics. However, the percent of angiogram-negative SAH in spontaneous SAH is 18.6% (208/1119), which is consistent with a previous study [17]. Besides, all angiogram-negative SAH patients were confirmed via CTA and DSA, and a repeated DSA was performed either 10–14 days after admission or one month after discharge to prevent misdiagnosis of angiogram-negative SAH. Second, potential bias may also exist in the definition of DCI and radiological scores. Additionally, it was difficult to assess the symptomatic vasospasm in patients with poor grade on admission. To limit such problems, we defined the DCI according to the criterion of previous studies [1, 7]. The radiological scales and DCI were independently evaluated by two senior neurologists who were blind to the clinical information. The DCI rate (5.8%) and poor outcome rate (mRS 3-6, 10.5%) of our study were also consistent with the past review reported (DCI: 0-9.6%; mRS 3-6: 0-12.6%) [17]. Second, there were also other studies that used different dichotomization of mRS or Glasgow Outcome Scale to assess the outcome, which may also introduce some bias to the performance of scales [18]. To limit this bias, we used two mRS dichotomization to define the poor outcome and increase the comparability to other studies. Nevertheless, the other outcome measures, such as in-hospital motility, mobility, and other prognostic scales or long-term outcomes should also be investigated. Third, the number of patients is relatively small, consecutive, and nonrandomized in our study, which may reduce the power and validity of the results. To avoid the confounding effect when comparing each scale, patients were excluded if they had serious comorbidities or sought care more than 3 days after SAH onset. Despite this consideration, a multicenter collaborative prospective validation with an increased cohort size is still recommended in future studies.

## 5. Conclusion

We have shown that VASOGRADE, HAIR, and WFNS may be a promising prognostic tool for predicting both DCI and poor outcome (mRS 3-6 and mRS 4-6). The mFS can be applicable for predicting DCI, poor outcome (mRS 4-6). The SAH score and HH were also optimal for predicting poor outcome. Conversely, SEBES appears inferior in predicting outcome.

## Figures and Tables

**Table 1:** Baseline characteristics.

		*n* = 190
Age (year, mean ± SD)		57.5 ± 11.8

Sex (male, %)		96 (50.5)

Smoking (%)		47 (24.7)

Drinking (%)		32 (16.8)

Hypertension (%)		57 (30.0)

Hyperlipidemia (%)		68 (35.8)

Diabetes (%)		12 (6.3)

Heart disease (%)		5 (2.6)

WFNS (%)	**1**	165 (86.8)
	**2**	8 (4.2)
	**3**	5 (2.6)
	**4**	2 (1.1)
	**5**	10 (5.3)

HH (%)	**1**	55 (28.9)
	**2**	112 (58.9)
	**3**	11 (5.8)
	**4**	6 (3.2)
	**5**	6 (3.2)

mFS (%)	**0**	21 (11.1)
	**1**	74 (38.9)
	**2**	45 (23.7)
	**3**	21 (11.1)
	**4**	29 (15.3)

SEBES (%)	**0**	107 (56.3)
	**1**	23 (12.1)
	**2**	23 (12.1)
	**3**	12 (6.3)
	**4**	25 (13.2)

VASOGRADE	**Green**	137 (72.1)
	**Yellow**	41 (21.6)
	**Red**	12 (6.3)

HAIR	**0**	91 (47.9)
	**1**	65 (34.2)
	**2**	23 (12.1)
	**3**	4 (2.1)
	**4**	1 (0.5)
	**5**	3 (1.6)
	**6**	2 (1.1)
	**7**	1 (0.5)

SAH score	**0**	37 (19.5)
	**1**	83 (43.7)
	**2**	39 (20.5)
	**3**	16 (8.4)
	**4**	9 (4.7)
	**5**	4 (2.1)
	**6**	1 (0.5)
	**7**	1 (0.5)

LOS (day, mean ± SD)		9.8 ± 8.2

Complications (%)	*DCI*	11 (5.8)
	*Hydrocephalus*	15 (7.9)
	*Rebleeding*	0
	*Seizure*	0

Outcome (mRS, %)	**0**	78 (41.1)
	**1**	62 (32.6)
	**2**	30 (15.8)
	**3**	7 (3.7)
	**4**	1 (0.5)
	**5**	3 (1.6)
	**6**	9 (4.7)
	*Unfavorable outcome* (**3-6**)	20 (10.5)
	*Unfavorable outcome* (**4-6**)	13 (6.8)

Abbreviations: DCI—delayed cerebral ischemia; HH—Hunt-Hess; LOS—length of hospital stay; mFS—modified Fisher Scale; mRS—modified Rankin Scale; SAH—subarachnoid hemorrhage; SD—standard error; SEBES—Subarachnoid hemorrhage Early Brain Edema Score; WFNS—World Federation of Neurosurgical Societies.

**Table 2:** OR and AUC of each score for predicting DCI and poor outcome in angiogram-negative patients.

	OR	95% CI	*P* for trend	AUC	95% CI
For predicting DCI					
WFNS	2.170	1.493-3.154	**0.001**	**0.773**	0.707-0.831
HH	2.431	1.461-4.044	**0.001**	0.677	0.606-0.743
mFS	3.193	1.684-6.053	**<0.001**	**0.839**	0.779-0.888
SEBES	1.587	1.092-2.307	**0.015**	0.696	0.625-0.760
VASOGRADE	6.916	2.840-16.844	**<0.001**	**0.858**	0.800-0.904
HAIR	1.849	1.315-2.601	**<0.001**	**0.809**	0.746-0.863
SAH score	1.735	1.192-2.525	**0.004**	0.710	0.640-0.773
For predicting poor outcome (mRS 3-6)					
WFNS	3.810	2.375-6.113	**<0.001**	**0.807**	0.744-0.861
HH	4.632	2.595-8.302	**<0.001**	**0.774**	0.708-0.831
mFS	1.813	1.242-2.645	**0.002**	0.712	0.643-0.776
SEBES	1.336	1.002-1.781	**0.049**	0.629	0.556-0.697
VASOGRADE	11.524	7.071-26.940	**<0.001**	**0.823**	0.761-0.874
HAIR	3.081	1.906-4.981	**<0.001**	**0.833**	0.772-0.883
SAH score	2.846	1.901-4.262	**<0.001**	**0.806**	0.742-0.860
For predicting poor outcome (mRS 4-6)					
WFNS	3.686	2.346-5.792	**<0.001**	**0.865**	0.808-0.910
HH	4.963	2.681-9.186	**<0.001**	**0.805**	0.741-0.858
mFS	2.439	1.466-4.058	**0.001**	**0.789**	0.724-0.845
SEBES	1.631	1.151-2.313	**0.006**	0.711	0.641-0.775
VASOGRADE	17.015	5.651-51.234	**<0.001**	**0.879**	0.824-0.922
HAIR	3.385	2.000-5.742	**<0.001**	**0.861**	0.803-0.907
SAH score	2.806	1.799-4.374	**<0.001**	**0.829**	0.768-0.880

Abbreviation: AUC—area under the curve; CI—confidence interval; DCI—delayed cerebral ischemia; HH—Hunt-Hess; mFS—modified Fisher Scale; mRS—modified Rankin Scale; OR—odd ratio, SAH——subarachnoid hemorrhage; SEBES—Subarachnoid hemorrhage Early Brain Edema Score; WFNS—World Federation of Neurosurgical Societies.

**Table 3:** Comparison of AUCs in angiogram-negative patients.

	*P* value for predicting DCI	*P* value for predicting mRS 3-6	*P* value for predicting mRS 4-6
Clinical scales			
WFNS vs. HH	0.070	0.354	0.150
Radiological scales			
mFS vs. SEBES	0.141	0.220	0.278
Combined scales			
VASOGRADE vs. HAIR	0.509	0.852	0.778
VASOGRADE vs. SAH score	0.077	0.781	0.474
HAIR vs. SAH score	0.084	0.479	0.354
Clinical vs. radiological scales			
WFNS vs. mFS	0.406	0.178	0.238
WFNS vs. SEBES	0.509	**0.022**	0.081
HH vs. mFS	0.195	0.430	0.826
HH vs. SEBES	0.900	0.078	0.351
Combined vs. single scales			
VASOGRADE vs. WFNS	0.189	0.646	0.686
VASOGRADE vs. HH	0.099	0.331	**0.029**
VASOGRADE vs. mFS	0.531	0.070	0.059
VASOGRADE vs. SEBES	0.110	**0.013**	**0.034**
HAIR vs. WFNS	0.547	0.521	0.914
HAIR vs. HH	0.141	0.313	0.448
HAIR vs. mFS	0.706	0.072	0.403
HAIR vs. SEBES	0.380	**0.009**	0.141
SAH score vs. WFNS	0.380	0.969	0.386
SAH score vs. HH	0.729	0.584	0.729
SAH score vs. mFS	0.180	0.238	0.640
SAH score vs. SEBES	0.925	**0.048**	0.141

Abbreviations: DCI—delayed cerebral ischemia; HH—Hunt-Hess; mFS—modified Fisher Scale; mRS—modified Rankin Scale; SAH—subarachnoid hemorrhage; SEBES—Subarachnoid hemorrhage Early Brain Edema Score; WFNS—World Federation of Neurosurgical Societies.

## Data Availability

The data used to support the findings of this study are available from the corresponding authors upon request.

## References

[1] Al-Mufti F., Merkler A. E., Boehme A. K. (2018). Functional outcomes and delayed cerebral ischemia following nonperimesencephalic angiogram-negative subarachnoid hemorrhage similar to aneurysmal subarachnoid hemorrhage. *Neurosurgery*.

[2] Jung J. Y., Kim Y. B., Lee J. W., Huh S. K., Lee K. C. (2006). Spontaneous subarachnoid haemorrhage with negative initial angiography: a review of 143 cases. *Journal of Clinical Neuroscience*.

[3] Rosen D. S., Macdonald R. L. (2005). Subarachnoid hemorrhage grading scales: a systematic review. *Neurocritical Care*.

[4] Hunt W. E., Hess R. M. (1968). Surgical risk as related to time of intervention in the repair of intracranial aneurysms. *Journal of Neurosurgery*.

[5] Oshiro E. M., Walter K. A., Piantadosi S., Witham T. F., Tamargo R. J. (1997). A new subarachnoid hemorrhage grading system based on the Glasgow Coma Scale: a comparison with the Hunt and Hess and World Federation of Neurological Surgeons Scales in a clinical series. *Neurosurgery*.

[6] Frontera J. A., Claassen J., Schmidt J. M. (2006). Prediction of symptomatic vasospasm after subarachnoid hemorrhage: the modified Fisher Scale. *Neurosurgery*.

[7] Ahn S. H., Savarraj J. P., Pervez M. (2018). The Subarachnoid hemorrhage Early Brain Edema Score predicts delayed cerebral ischemia and clinical outcomes. *Neurosurgery*.

[8] de Oliveira Manoel A. L., Jaja B. N., Germans M. R. (2015). The VASOGRADE: a simple grading scale for prediction of delayed cerebral ischemia after subarachnoid hemorrhage. *Stroke*.

[9] Lee V. H., Ouyang B. C., John S. (2014). Risk stratification for the in-hospital mortality in subarachnoid hemorrhage: the HAIR score. *Neurocritical Care*.

[10] Naval N. S., Kowalski R. G., Chang T. R., Caserta F., Carhuapoma J. R., Tamargo R. J. (2014). The SAH score: a comprehensive communication tool. *Journal of Stroke and Cerebrovascular Diseases*.

[11] Connolly E. S., Rabinstein A. A., Carhuapoma J. R. (2012). Guidelines for the management of aneurysmal subarachnoid Hemorrhage. *Stroke*.

[12] Diringer M. N., Bleck T. P., Claude Hemphill J. (2011). Critical care management of patients following aneurysmal subarachnoid hemorrhage: recommendations from the Neurocritical Care Society’s Multidisciplinary Consensus Conference. *Neurocritical Care*.

[13] Frontera J. A., Fernandez A., Schmidt J. M. (2009). Defining vasospasm after subarachnoid hemorrhage: what is the most clinically relevant definition?. *Stroke*.

[14] de Haan R., Limburg M., Bossuyt P., van der Meulen J., Aaronson N. (1995). The clinical meaning of Rankin “handicap” grades after stroke. *Stroke*.

[15] Dengler N. F., Sommerfeld J., Diesing D., Vajkoczy P., Wolf S. (2018). Prediction of cerebral infarction and patient outcome in aneurysmal subarachnoid hemorrhage: comparison of new and established radiographic, clinical and combined scores. *European Journal of Neurology*.

[16] Zhou X. H. (1998). Comparing correlated areas under the ROC curves of two diagnostic tests in the presence of verification bias. *Biometrics*.

[17] Kapadia A., Schweizer T. A., Spears J., Cusimano M., Macdonald R. L. (2014). Nonaneurysmal perimesencephalic subarachnoid hemorrhage: diagnosis, pathophysiology, clinical characteristics, and long-term outcome. *World Neurosurgery*.

[18] St Julien J., Bandeen-Roche K., Tamargo R. J. (2008). Validation of an aneurysmal subarachnoid hemorrhage grading scale in 1532 consecutive patients. *Neurosurgery*.

